# Identification of a novel *CHEK2 *variant and assessment of its contribution to the risk of breast cancer in French Canadian women

**DOI:** 10.1186/1471-2407-8-239

**Published:** 2008-08-15

**Authors:** David J Novak, Long Qi Chen, Parviz Ghadirian, Nancy Hamel, Phil Zhang, Vanessa Rossiny, Guy Cardinal, André Robidoux, Patricia N Tonin, Francois Rousseau, Steven A Narod, William D Foulkes

**Affiliations:** 1 Program in Cancer Genetics, Departments of Oncology and Human Genetics McGill University, Montreal, QC, H2W 1S6, Canada; 2 Departments of Medicine and Human Genetics, McGill University, Montreal, QC, H2W 1S6, Canada; 3 Segal Cancer Center, McGill University Sir M. B. Davis-Jewish General Hospital, 3755 Cote Ste-Catherine, Montreal, QC, H3T 1E2, Canada; 4 Department of Thoracic and Cardiovascular Surgery, West China Hospital, Sichuan University, Chengdu 610041, PR China; 5 Epidemiology Research Unit, Research Centre, Centre Hospitalier Université de Montréal, 3850 St-Urbain, Montréal, QC, H2W 1T7, Canada; 6 The Research Institute, McGill University Health Centre, 1650 Cedar Avenue, Montréal, QC, H3G 1A4, Canada; 7 Women's College Research Institute, University of Toronto, 790 Bay Street, Toronto, ON, M5G 1N8, Canada; 8 The CanGènetest Research Consortium on genetic Laboratory Services, Centre de recherché du CHUQ/HSFA, 10 rue de l'Espinay, Québec, QC, G1L 3L, Canada; 9 Centre de recherché du CHUQ – Hôpital St-François d'Assise, Centre Hospitalier Université de laval, 10 rue de l'Espinay, Québec, QC, G1L 3L5, Canada; 10 Department of Surgery, Centre Hospitalier Université du Montréal, 3850 St-Urban, Montréal, QC, H2W 1T7, Canada

## Abstract

**Background:**

*BRCA1 *and *BRCA2 *account for the majority of the known familial breast cancer risk, however, the impact of other cancer susceptibility genes largely remains to be elucidated. Checkpoint Kinase 2 (*CHEK2*) is an important signal transducer of cellular responses to DNA damage, whose defects have been associated with an increase in breast cancer risk. Previous studies have identified low penetrance *CHEK2 *alleles such as 1100delC and I157T, as well as variants such as S428F in the Ashkenazi Jewish population and IVS2 + 1G>A in the Polish population. No founder allele has been specifically identified in the French Canadian population.

**Methods:**

The 14 coding exons of *CHEK2 *were fully sequenced for variant alleles in a panel of 25 affected French Canadian women and 25 healthy controls. Two variants were identified of which one novel variant was further screened for in an additional panel of 667 breast cancer patients and 6548 healthy controls. Additional genotyping was conducted using allele specific PCR and a restriction digest assay. Significance of amino acid substitutions were deduced by employing comparative analysis techniques.

**Results:**

Two variants were identified: the previously reported silent substitution 252A>G (E84E) and the novel missense variant, 1217G>A (R406H). No significant difference in allele distribution between French Canadian women with breast cancer and healthy controls was observed (3/692, 0.43% vs. 22/6573, 0.33%, respectively, P = 0.73).

**Conclusion:**

The novel CHEK2 missense variant identified in this study, R406H, is unlikely to contribute to breast cancer risk in French Canadian women.

## Background

Breast cancer is the most common form of malignancy amongst females in the western world. Specifically, one in ten of all new diagnosed cancer cases are of the female breast [1]. Typically, less than five percent of these cases are inherited in a mendelian fashion, specifically from the segregation of highly penetrant alleles, such as mutations in *BRCA1 *and *BRCA2 *[2]. The existence of a large number of breast cancer families who lack linkage to either *BRCA1 *or *BRCA2 *[3] suggested that other breast cancer susceptibility genes remained undiscovered. One such candidate gene, CHEK2, encodes a multifunctional kinase enzyme involved in the induction of cell cycle arrest, DNA repair and apoptosis [4-6]. Several large-scale studies have characterized known variants of the CHEK2 gene [7-9], conclusively proving that CHEK2 is a breast cancer susceptibility gene.

One *CHEK2 *mutation present in the general population, 1100delC, occurs independently of *BRCA1/2 *mutations [7,8]. The 1100delC variant results in a premature stop codon within exon 10, impairing the kinase ability of the enzyme and resulting in a two-fold increase in breast cancer risk [7,8,10]. In general, the population frequency of 1100delC has been reported to be ~1.9% in individuals with breast cancer, compared to ~0.7% in those without [10]. There is, however, variation in the observed frequency of 1100delC [10-13] suggesting that the prevalence of this mutation varies amongst populations.

Population isolates, also known as founder populations, have reduced genetic heterogeneity and are valuable tools for genetic analysis involving cancer susceptibility. A recent example of such an approach has been seen with the identification of the *CHEK2 *S428F mutation in the Ashkenazi Jewish population, which has been associated with a relative breast cancer risk of 2.0 amongst Ashkenazi Jewish women [14]. Similarly, a splice site mutation, IVS2 + 1G>A, originally identified in a US patient with familial prostate cancer [15], has been identified as a founder mutation in the Polish population with a population frequency of 0.3% [16]. The allele is associated with a two- to four-fold elevated risk for prostate, as well as a moderate increase in risk for breast cancer [16,17]. Most recently, Walsh et al. [18] discovered a novel 5.4 Kb deletion, leading to a loss of exons 9 and 10, in two families of Central European ancestry. This mutation was found in 1.3% of 631 patients and in none of the 367 healthy controls. Further analysis of *CHEK2 *may reveal additional founder mutations in other populations. One such population yet to be investigated, and the focus of this study, is the French Canadian population.

Established in Quebec between 1608 and 1760, the population now includes approximately 6 million French Canadians, who are descendants of an estimated 8000–10000 migrants from France [19]. Altogether, approximately 80% of these founders still have descendants in Quebec today, and they account for the major part of the French Canadian gene pool [20]. Many of the hereditary disorders in the French Canadian population show evidence of founder effects (for review, see [19]). In particular, French Canadian founder mutations have been identified in *BRCA1*, *BRCA2 *and *PALB2 *[21-24].

In the current study, we examined a panel of 25 *BRCA1/2 *negative, affected French Canadian women alongside 25 healthy controls, to investigate the impact of *CHEK2 *variants on breast cancer susceptibility in the French Canadian population.

## Methods

### Study Population

French Canadian women, previously affected by breast cancer, and determined through sequencing to be negative for all exonic *BRCA1 *and *BRCA2 *mutations, were used for SNP discovery (n = 25). Cases had a family history of breast cancer with at least three cases of either breast cancer diagnosed before 65 years of age, male breast cancer, or ovarian cancer within three degrees from the index case [21]. Healthy French Canadian women with unknown *BRCA1/2 *mutation status were used as controls (n = 25). Controls were requited either through random dialing or as spouses of cases ascertained for previous studies of cancer, in the French Canadian population (**Group 1**, n = 50).

Variants identified in the initial case/control group were further screened for in extended groups of breast cancer cases and unaffected controls, using the original carrier samples as a positive control. **Group 2 **consists of cases (n = 124) which were tested, and found negative, for French Canadian *BRCA1/2 *mutations reported by Tonin et al [21]. Women included in this group were diagnosed at a mean age of 54 (range = 26–76) years old and were referred to cancer genetics clinics at McGill University hospitals. Patients included in Group 2 were selected for either a high risk family history of at least three cases of breast and/or ovarian cancer within three degrees from the index case, or for presentation of multiple consecutive breast cancer cases prior to the age of 76. Cases included in this panel were genotyped alongside a subset of healthy French Canadian women, recruited through random dialing, in the clinic or as spouses of cases from previous investigations, as controls (n = 116). **Group 3 **includes an extended group of French Canadian women (n = 543) previously diagnosed with breast cancer at Hotel-Dieu Hospital, Montreal, at a mean age of 47 (range = 26–65) years old. All women in this group had previously been tested and found negative for French Canadian *BRCA1/2 *founder mutations. Recruited patients were either under 50 years of age at diagnosis, or were diagnosed between 50 and 65 and had a first degree relative with breast cancer. **Group 4 **consists of a panel of French Canadian neonatal controls (n = 6432), which have been previously tested for several known *PALB2 *variants [24] as well as the known *BRCA1 *and *BRCA2 *French Canadian founder mutations.

All patients have provided written consent to participate in current research based investigations. The study is in compliance with the Helsinki declaration, and has been granted ethical approval by the institutional review boards of McGill University and the University of Toronto.

### Molecular methods

#### Genotyping

SNP discovery was performed on Group 1 by direct PCR and sequencing (sequencing was conducted by the *McGill University and Genome Quebec Innovation Center *in both the forward and reverse directions). Sequencing was performed on all of the 14 coding exons of *CHEK2 *as well as at the intron/exon boundaries. Primers used for PCR were designed using the online Primer3 program (Primer3). All primers used, annealing temperatures and amplicon sizes are summarized in Table 1.

**Table 1:** CHEK2 Primers and Details

**Fragment**	**Size (bp)**	**Exon**	**Amino Acid**	**Primers (5'->3')**	**Annealing ** **Temp.(°C)**
CHEK2EX01	565	1	1–106	Forward: gaactataggtctgggctgttagg Reverse: tccacctggtaatacaactttctg	57
CHEK2EX02	582	2&3	107–197	Forward: tgccttcttaggctattttcctac Reverse: aaccatattctgtaaggacaggac	56
CHEK2EX04	354	4	198–228	Forward: ctcaagggctttacaatatg Reverse: gaaatgagaaaccaccaatc	54
CHEK2EX05	499	5	229–264	Forward: gaatttcacaatccagggctac Reverse: ctcacaaattcatccatctaagcag	56
CHEK2EX06	632	6	265–282	Forward: tagagctgggtttggaactcag Reverse: agctaggcatgtgtgtgaatg	68
CHEK2EX07	434	7	283–304	Forward: aagaagactgggaagagacctagc Reverse: gcaagcctacattagattctttgg	56
CHEK2EX08	365	8	305–336	Forward: catctcattccttagtttccaactg Reverse: tctgcctaattcagggagtaattc	56
CHEK2EX09	331	9	337–365	Forward: ctgtgagatgtgtgtgttggtaac Reverse: tctggataagagcagtatcacctg	58
CHEK2EX10	546	10	366–420	Forward: ttaatttaagcaaaattaaatgtcc Reverse: ggcatggtggtgtgcatc	54
CHEK2EX11	353	11	421–458	Forward: gctgggattacaagcctaagg Reverse: gaagaaactcccaccacagc	69
CHEK2EX12	541	12	459–487	Forward: ggcctgttaattctggcatactc Reverse: aaaggttgtagcctggccag	67
CHEK2EX13	488	13	488–514	Forward: cctctgggaaggtagaggc Reverse: caatccctagctgtgcttatcg	66
CHEK2EX14	585	14	515–543	Forward: cccccactttactggaagc Reverse: gcaaaaccctgtctctacaaaat	64
CHEK2 R406H Allele Specific	N/A	10	N/A	Forward: ggactgctgggtataacca	54
CHEK2 Long Range	~9,200	10–14	366–543	Forward: cgacggccagtctcaagaagaggactgtctt Reverse: gctatgaccatgcacaaagcccaggttccatc	58
CHEK2 Restriction	546	10	366–420	Forward : ttaatttaagcaaaattaaatgtc Reverse : ggcatggtggtgtgcatc	57
CHEK2 Restriction Nested	202	10	380–420	Forward: catgagaaccttatgtggaaccc Reverse: cctggacaacagagcaagacacat	58
CHEK2 1100delC Sizing	196	10	366–396	Forward:aatagaaactgatctagcctacgtgt Reverse: gaacttcaggcgccaagt	60

Summary of primers, annealing termperatures and PCR amplicon sizes for the 14 coding exons of CHEK2. Additional details are listed for primers used for Long Range PCR, R406H and 1100delC genotyping.

#### Long Range PCR

Any variants found within exons 10–14, which are known to be duplicated wholly or in part on various chromosomes, were reamplified via long range PCR; a ~9.2 Kb fragment encompassing exons 10–14 was generated using primers F5'-CGACGGCCAGTCTCAAGAAGAGGACTGTCTT-3' and R5'-GCTATGACCATGCACAAAGCCCAGGTTCCATC-3' as previously described [14]. PCR was conducted using the Expand Long Template PCR system (Roche Applied Science, Cat No. 1-681-834) with an annealing temperature of 58°C.

Products obtained from Long-range PCR were then used as a template in a second round of amplification, using appropriate primers to isolate individual exons for sequencing.

#### Allele-Specific PCR

To determine the frequency of 1217G>A in Group 2, a forward primer with the last nucleotide specific to the variant was designed and used in conjunction with the exon 10 primers designed for sequencing. PCR was conducted at an annealing temperature of 54°C and the product was visualized by gel electrophoresis.

Allele-specific amplification was preformed as above for Group 4 which was followed by fluorometric detection of the PCR product using SybrGreen. A scatter plot was derived from the raw fluorescence of both alleles which was then analyzed to compute the genotype as previously described [25]. The accuracy of this method is 99.0% and the average rate of data rejection is below 1.00%.

#### Restriction Assay

Samples from Group 3 were genotyped via a restriction digest assay. Samples were amplified by PCR twice: the first to isolate *CHEK2 *exon 10, and the second using nested primers to obtain a smaller fragment of 202 bp, encompassing 1217G>A. Products obtained from the second round of amplification were incubated overnight at 37°C with +*Nla*III (1 U/sample, New England BioLabs, USA). NlaIII digests after the consensus sequence of CATG, and thus cut the variant (A) allele, resulting in three fragments of 4, 76 and 122 bp, respectively. After digest, the wildtype CHEK2 allele results in two fragments of 4 and 198 bp, respectively. A sample mutant for R406H (confirmed by sequencing) and a wild-type sample were randomly seeded on each 96-well plate and used as positive and negative controls respectively in the screening process. Digested products were visualized by gel electrophoresis. The presence of 1217G>A was confirmed by direct sequencing using the BigDye^® ^Terminator v1.1 Cycle Sequencing Kit and 3130 × l Genetic Analyzer (Applied Biosystems, USA).

#### 1100delC mutation Analysis

The presence of 1100delC within samples encompassing Group 2 was determined by generating S-35 labeled PCR products. PCR product was denatured for 15 min at 95°C prior to loading in a 5% denaturing polyacrylamide gel. PCR products were separated for 2 hours at 80W and visualized by audioradiography.

### Amino Acid stability, conservation and severity

To estimate the impact of amino acid substitutions on phenotype, mean chemical distance between the wild type amino acid and its substitute was evaluated using the Grantham matrix score (Grantham, 1974), Grantham variation (GV) and Grantham deviation (GD). Conservation of the wild type amino acid was analyzed using the multiple sequence alignment program ClustalW. Substitution tolerance was estimated using the SIFT algorithm (Sorting Intolerant From Tolerant).

### Statistical analysis

Allele and genotype frequency is expressed as a proportion of the entire sample set. Fisher's exact test was used to test for significance. In the circumstance where a sample would not amplify, it was excluded from all calculations. Two-tailed p values are presented.

## Results

**Figure 1:**
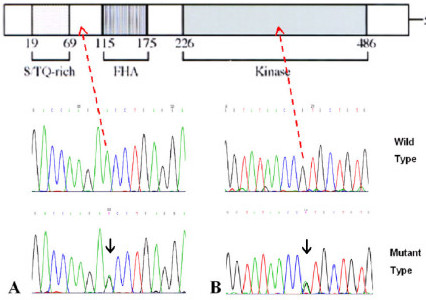
**E84E and R406H**. A) Chromatogram of the silent E84E with arrow illustrating its location N' Terminal to the CHEK2 fork-head association domain. B) Chromatogram of R406H and its location within the CHEK2 Kinase domain.

SNP discovery in *CHEK2 *coding regions was conducted by sequencing 25 cases and 25 controls simultaneously. This approach provides an 80% power to detect an allele with a frequency of 1% or more [26]. Furthermore, this eliminates the potential biases inherent when studying cases first and then searching for only those variants identified, in the control set. From this, we have identified two variants: the previously reported silent variant, 252A>G (E84E), observed in 2/25 cases versus 2/25 controls, in addition to the novel missense variant 1217G>A, which results in an amino acid substitution at position 406, of an arginine for a histidine (R406H, Figure 1) observed in 1/25 cases.

The missense mutation, R406H was further screened for in extended groups of cases and controls. Through allele-specific PCR, we identified one additional affected case (1/124, 0.81%) from Group 2. Group 3 was genotyped by a restriction assay and was found to contain one affected case (1/543, 0.18%). Within our neonatal set of controls, Group 4, R406H was observed in 22 samples (22/6432, 0.34%). Overall, the frequency of the R406H allele was not significantly elevated in total breast cancer cases (3/692, 0.43%) compared with healthy controls (22/6573, 0.33%) P = 0.73 (Table 2).

**Table 2:** CHEK2 1217G>A Frequency

**Group**	**BRCA**	**CTRL**	**P-Value**
**1**	4.00% (1/25)*	0.00% (0/25)*	1.00
**2**	0.81% (1/124)*	0.00% (0/116)*	1.00
**3**	0.18% (1/543)	N/A	N/A
**4**	N/A	0.34% (22/6432)	N/A

**Total**	**0.43% (3/692)**	**0.33% (22/6573)**	**0.73**

*Genotyped for 1100delC which was observed in 2.01% (3/149) of cases vs 0.7% (1/141) controls. If we compare the frequency in cases with that seen in the same neonatal controls used in this study, that were also tested for 1100delC by Zhang et al [30] (19 1100delC carriers among 6460 controls), then the difference between cases and controls is statistically significant (*P *= 0.01).

To predict the significance of the R406H substitution, sequence alignment of *CHEK2 *exon 10 was analyzed across ten species, revealing a modest conservation of the arginine residue amongst higher eukaryotes, with 6/10 species displaying homology (Table 3). When comparing the mean chemical difference between arginine and histidine, a Grantham score of 29, GV of 124.29 and a GD of 0.0 is obtained, suggesting the neutrality of this substitution. Furthermore, tolerance of this substitution is indicated via analysis by the SIFT algorithm (SIFT score of 0.10).

**Table 3:** Sequence Alignment of *CHEK2 *Exon 10

Mosquito	VSDFGSSKFLDHTIFMRTICGTPEYVAPEVLESNGQKPYT**R**QVDVWSLGVVLYTM --256
Fruit Fly	VSDFGLSKFVQKDSVMRTLCGTPLYVAPEVLITGGREAYTKKVDIWSLGVVLFTC --376
Homo Sapiens	ITDFGHSKILGETSLMRTLCGTPTYLAPEVLVSVGTAGYN**R**AVDCWSLGVILFIC --420
Chimpanzee	
Dog	ITDFGQSKILGETSLMRTLCGTPTYLAPEVLNSFGTAGYN**R**AVDCWSLGVILFIC --421
Mouse	ITDFGQSKILGETSLMRTLCGTPTYLAPEVLVSNGTAGYS**R**AVDCWSLGVILFIC --424
Rat	ITDFGQSKILGETSLMRTLCGTPTYLAPEVLISNGTAGYS**R**AVDCWSLGVILFIC --423
Chicken	-TYFGQSKILGETSLMKTLCGTPTYLAPEVLNSFGTAGYS**R**AVDCWSLGVILFVC --391
Fugu	VTDFNQSRILEETMLMRTLCGTPSYLAPEVFTQASTTGYSLAVDAWSLGVLLFVC --396
Tetraodon	VTDFNQSRILEETMLMRTLCGTPSYLAPEVFTQASTSGYGLAVDAWSLGVLLFVC --430
C. Elegans	LTDFGMAKNSVN--RMKTHCGTPSYCAPEIVANQG-VEYTPKVDIWSLGCVLFIT --370

Additionally, patients included in Group 2 were further genotyped for 1100delC. Including the fully sequenced 25 cases and controls, 1100delC was observed in 2.01% (3/149) of cases versus 0.7% (1/141) of controls.

## Discussion

Inherited breast cancer has been associated with germline mutations in more than ten different genes, most of which are involved in the maintenance of genomic integrity. A large proportion of such cases can be accounted for by mutations in the tumor suppressor genes *BRCA1 *and *BRCA2*. Additionally, *TP53*, *PTEN*, *CDH1 *and *STK11 *are considered high-risk breast cancer susceptibility genes. Mutations in *ATM, BRIP1, PALB2, CHEK2 *and possibly *NBS1, RAD50 *are also associated with a moderately increased risk for breast cancer, and many low penetrance genes have recently been identified. However, roughly 50% of familial breast cancers remain to be elucidated [27,28].

In the current study, 25 French Canadian breast cancer patients and 25 healthy controls were fully screened for variants within the *CHEK2 *gene. Two variants were identified: the silent variant E84E and the novel R406H missense variant. E84E, which has been reported in several other *CHEK2 *screens, is likely a neutral allele with no association to breast cancer [14,29,30]. In addition, given that the primary structure of CHEK2 is unaltered by the E84E mutation, and further, that it was observed at a similar frequency in cases and controls suggests against the possibility that this variant may affect an exonic splicing enhancer or aberrantly affect protein translation rates. Thus, no further investigation of this variant was conducted. R406H, however, was genotyped for in an extended panel of breast cancer cases and healthy controls. Neither variant was observed at a significantly high frequency in breast cancer cases when compared with controls.

To further characterize any potential impact of R406H, bioinformatic tools were employed. In short, conservation analysis, substitution evaluation and a tolerance test lack any indication of a pathogenic contribution from this allele.

Large international studies [10,31-33] have shown that 1100delC is associated with increased breast cancer risk in many, but by no means all, world populations. Our findings in cases (Table 2) when combined with previous data on controls [32] suggest that this allele is also associated with breast cancer risk in the French Canadian population. The evidence that other *CHEK2 *alleles are associated with an increased risk in the general population is less convincing [34,35]. However, some founder alleles that do seem to be associated with an increased risk in specific populations have been identified.

To date, five interesting *CHEK2 *founder alleles have been identified, all of which are associated with an elevated risk for breast: 1100delC, I157T, IVS2 + 1G>A, S428F and del5395. All five variants have been shown to contribute to breast cancer risk provided they are present in the population of interest, with the latter three particularly being observed with high degree of ethnic specificity. The IVS2 + 1G>A splicing mutation has been observed in the Polish population as a founder mutation with a 0.3% population frequency [36] and associates with approximately a two-fold elevated risk for breast cancer. In the Ashkenazi Jewish population, Shaag et al [14] discovered the novel missense mutation S428F (1283C>T) at a frequency of 2.88% amongst 1632 breast cancer patients compared to 1.37% of 1673 controls, thus suggesting S428F is associated with breast cancer risk; a yeast complementation assay supported the hypothesis that this variant aberrantly affects *CHEK2 *protein function. The most recently identified founder mutation, del5395, resulting in a loss of exons 9 and 10, was originally identified in two families of Czech or Slovak origin [18]. This founder mutation has twice been studied in detail; the first observing the deletion in 1.3% of 631 breast cancer cases and 0.0% of 367 healthy controls from the Czech and Slovak Republics. In agreement with the first study, Cybulski et al [37] investigated the 5,395 bp deletion in Poland, observing the frequency to be 0.9% of 4,454 breast cancer cases versus 0.4% of 5,496 healthy controls (OR = 2.0; 95% CI = 1.2–3.4). It is likely other *CHEK2 *founder mutations are yet to be discovered, as to date, *CHEK2 *has not been thoroughly investigated in many ethnic groups.

One such group, the French Canadian population has proved to be valuable in investigations of other breast cancer susceptibility genes. For example, several common pathogenic *BRCA1/2 *founder mutations are recognized in the French Canadian population [21-23]. Moreover, the proposition that additional French Canadian founder mutations have yet to be revealed is supported by the recent identification of a *PALB2 *truncating mutation, Q775X [24].

The results presented here represent the first systematic analysis of *CHEK2 *in the French Canadian population. The novel variant we identified, R406H, is almost certainly not associated with increased risk for breast cancer and *CHEK2 *alleles other than 1100delC are unlikely to contribute to breast cancer risk in this population. However, the possibility that *CHEK2*, due to its role in cell cycle regulation, may influence the risk of other familial cancers in the French Canadian population, such as prostate, colon, ovarian or colorectal cancer, and would thus be an informative population for such future investigations. Interestingly, some of the well known variants, such as I157T have been associated with colon cancer [38], whereas the truncating variants 1100delC and IVS2 + 1G>A have been associated with an elevated risk for familial prostate cancer in both the Polish and Finish population [16]. Most recently, all three variants in addition to the del5395 have been associated with an increased susceptibility to bladder cancer in Poland [39].

The emerging picture suggests that some functionally significant variants in *CHEK2 *are able to predispose cells from a wide distribution of organs to an elevated risk of cancer. Thus, much remains to be studied with respect to *CHEK2 *alleles in the French Canadians, but it seems unlikely that a specific, common founder mutation for breast cancer exists in this population.

## Conclusion

Sequencing of the *CHEK2 *gene in 25 breast cancer patients and 25 healthy controls, from the French Canadian population did not reveal any pathogenic mutations. The one novel missense variant identified in this study, R406H, does not appear to be associated with breast cancer risk. Additional investigations of CHEK2 and French Canadian breast cancer, utilizing large panels of familial and/or sporadic cases, would be necessary to refute the notion that additional CHEK2 susceptibility alleles exist in the French Canadian population. However, it is unlikely that CHEK2 alleles other than 1100delC significantly influence familial breast cancer risk within our study group.

Note added in Proof: We have recently completed MLPA (MRC-Holland, kit P190) analysis on 41 French Canadian women with a personal and familial history breast cancer. Cases had previously been screened for all known founder *BRCA1 *and *BRCA2 *mutations, as well as CHEK2 1100delC. No genomic deletions or insertions were identified.

## Competing interests

The authors declare that they have no competing interests.

## Authors' contributions

Experimental design was conceived by DJN, LQC, NH and WDF. Data acquisition was conducted by DJN under the supervision of WDF. Initial technical optimizations were conducted by VR and NH. Sample recruitment and implementation was carried out in collaboration with PG, PT and AR. Neonatal genotyping was performed by GC and FR. Additional French Canadian R406H genotyping was carried out by SAN and PZ. DJN drafted the manuscript, which was revised by WDF. All authors have given their final approval of the version to be published.

## Pre-publication history

The pre-publication history for this paper can be accessed here:


